# Malignancy of Nose and Paranasal Sinuses: An Institutional Study in Central India

**DOI:** 10.7759/cureus.52074

**Published:** 2024-01-11

**Authors:** Anjan K Sahoo, Rohini R Nair, Utkal P Mishra, Ganakalyan Behera, Shaila Sidam, Vikas Gupta

**Affiliations:** 1 Otolaryngology - Head and Neck Surgery, All India Institute of Medical Sciences, Bhopal, IND

**Keywords:** squamous cell carcinoma (scc), head and neck sarcoma, sinonasal adenocarcinoma, nose, fess and endoscopic surgery for pns maligancies, paranasal sinus neoplasm

## Abstract

Introduction: Malignancy of the nose and paranasal sinuses is a highly heterogeneous tumor group that arises from various cell types commonly seen in the fifth to sixth decades of life, with twice as much commonness in males. Patients present with varied clinical presentations like nasal obstruction, facial swelling, orbital complications, etc. Squamous cell carcinoma and adenocarcinoma are the most common variant. Surgery followed by adjuvant chemo or radiotherapy is the treatment of choice.

Methods: The study was undertaken in the Department of Otorhinolaryngology All India Institute of Medical Sciences, Bhopal, India, from 2021 to 2023. It was a retrospective study in which patients diagnosed and underwent treatment in the last 2 years were enrolled. Data were retrieved from the medical record department and surgical registry. Twenty-eight patients were recruited for the study. Detailed history, clinical examination, imaging findings, surgical plans, postoperative adjuvant therapy details, and histopathological findings were recorded.

Results: There were 18 (64.2%) males and 10 (35.8%) females, with a male-to-female ratio of 1.8: 1. The mean age of patients was 50.5 years. Facial swelling was the most frequent symptom (n=15, 54%). Twenty-one (75%) patients use chewable tobacco, while sixteen (57%) are smokers. All our patients belong to the lower socioeconomic group. Endoscopic resection was done in 15 (62.5%) patients, and combined open and endoscopic approaches were used in 9 (37.5%) patients. The most common histological variant was squamous cell carcinoma (n=8, 28%).

Conclusion: Malignancy of the nose and paranasal sinus is very rare. They presented with varied masked clinical presentations of benign diseases. Early identification and high clinical suspicion, along with imaging studies, are pivotal in managing malignancy of the nose and paranasal sinuses.

## Introduction

Sinonasal tumors are a highly heterogeneous group of tumors accounting for less than 1% of all malignancies and about 3% of all head and neck malignancies [1]. These tumors can arise from various cell types, including epithelial, glandular, and mesenchymal cells. While rare, sinonasal malignancies are a significant clinical concern due to their challenging diagnosis and aggressive nature. They are seen in the fifth and sixth decades of life and are twice as common in males as females. Various factors, like occupational exposure to heavy metals such as nickel and chromium, leather, textile, and wood industries, along with tobacco and alcohol, predispose to the development of malignancies in the sinonasal tract [2].

Indian studies have shown that the incidence of sinonasal malignancies in India is approximately 1/100,000 persons per year [3]. The most common histological subtypes of sinonasal malignancy in India are squamous cell carcinoma and adenocarcinoma. These two subtypes together represent the majority of cases, with squamous cell carcinoma accounting for around 50% of sinonasal tumors and adenocarcinoma accounting for 13% [4]. Other less common histologies observed in Indian studies include sarcoma, undifferentiated carcinoma, teratocarcinosarcoma, malignant melanoma, adenoid cystic carcinoma, lymphoma, olfactory neuroblastoma, and neuroendocrine carcinoma.

Patients may present with vague symptoms such as nasal obstruction, nasal discharge, epistaxis, facial pain or swelling, anosmia, nasal discharge, and proptosis. Specialized imaging studies, such as computed tomography and magnetic resonance imaging, play a crucial role in diagnosing and staging sinonasal malignancies. Furthermore, histopathological examination of biopsy samples is essential to confirm the diagnosis and determine the specific subtype of sinonasal malignancy. Different factors delay the diagnosis of these rare tumors, like non-specific clinical manifestation, limited anatomical access, and a variety of histological types of tumors. A nearby critical structure like the skull base, optic nerve, carotid artery, cavernous sinus, and brainstem complicates surgery and rehabilitation [5]. Imaging has a significant role in the management of paranasal sinus malignancy. It helps to know the site of origin of the tumor and its extension to nearby structures. Surgery followed by adjuvant radio and chemotherapy, depending on the histopathology, is the treatment of choice for this type of tumor. Sinonasal tumors carry a poor prognosis despite the early diagnosis, radical surgical resection, and strict follow-up. In this study, we aimed at understanding the characteristics and prognosis of these tumors in the Indian population.

## Materials and methods

A single-center, hospital-based observational retrospective study was undertaken in the department of otorhinolaryngology of AIIMS Bhopal, India, for two years (2021-2023). All patients with a diagnosis of sinonasal malignancy presenting to the ENT department in the past two years and underwent surgery and adjuvant therapy as per the standard treatment guidelines were included in the study. Patients with secondary involvement of paranasal sinuses by tumors from distant sites and nasopharyngeal tumors were excluded from the study (Table 1).

**Table 1:** Inclusion and exclusion criteria

Inclusion Criteria	Exclusion Criteria
Patients with the diagnosis of the nose and paranasal sinus malignancy underwent surgery or adjuvant therapy as per the recommendation of the head and neck tumor clinic.	Cases where a complete set of data was not available in records.
Patients where complete information, including histopathological and radiological data, was available	Patients with secondary involvement of paranasal sinuses by tumors from distant sites and nasopharyngeal tumors

Institutional human ethics committee approval was taken before commencing the study. Twenty-eight patients were enrolled in the study. Data were retrieved from the surgical registry and the medical record department. The demographic and clinical profiles of these patients were recorded in a worksheet. A detailed history with particular reference to age, sex, residence, occupation, family history, past history, allergic disorders, and addictive habits, followed by a clinical examination with special reference to the nose and paranasal sinuses, was recorded from the patient's retrieved file and analyzed. All patients' age groups and relation to any particular type of malignancy were also correlated. Patients were also categorized as per their occupation, like farmers, people in business, and housemakers. Signs and symptoms, including common nasal findings like nasal obstruction, nasal discharge, nasal bleedings, and rare symptoms like cranial nerve palsies, ulceration in the hard palate, and skin over the maxilla, were also noted. Anterior nasal endoscopy findings, like characteristics of the mass and growth, were also retrieved. Probing of nasal mass and detailed digital examination of mass in the oral cavity and face were also noted. Imaging findings like CT and MRI were also noted. Various operative techniques like endoscopic, open, and combined approaches were also documented. Intraoperative and imaging results were compared and analyzed. Complications that occurred during surgery were also noted. The patients received adjuvant therapy as per their histological findings, and tumor staging was also reported. The eighth edition of the American Joint Committee on Cancer Staging Classification was used for most histological types except lymphoma and melanoma. Data was analyzed using Microsoft Office Excel 2010.

## Results

There were 18 (64.2%) males and 10 (35.8%) females, with a male-to-female ratio of 1.8: 1. The age ranges from 23 to 72 years, with a mean age of 50.5 years. The most frequent symptoms in our series were facial swelling seen in 15 (54%) patients (Table 2), followed by sinonasal undifferentiated carcinoma (SNUC) (n=6, 22%), Adenocarcinoma (n=3, 11%) and sarcoma (n=3, 11%). Recurrence was noticed in two patients; two patients died during follow-up, and one patient got maggot infection. Followed by nasal obstruction (n=12, 43%) and diplopia (n=12, 43%). Other symptoms were watering from the eye (n= 8, 28%), headache (n= 7, 25%), epistaxis (n=6, 21%), swelling in the hard palate (n=5, 18%), proptosis (n=4, 14%), loosening of the tooth (n=4, 14%), loss of vision (n=2, 7%), ulceration over hard palate (n=2, 7%), ulceration over facial skin (n=2, 7%). Most of the patients were in their sixth decades (n=8, 28%), followed by seventh decades (n=7, 25%) and fifth decades (n=4, 14%) of life. Sixteen (57%) patients were smokers. Twenty-one (75%) patients use chewable tobacco, nine (32%) patients regularly drink alcohol, and eight (28%) patients have the habit of both smoking and drinking. Most of our patients were farmers (n=15, 54%), followed by homemakers (n=8, 28%). All our patients belong to the lower socioeconomic group according to the modified Kuppuswamy socioeconomic scale. All the patients had a CT scan, MRI, or both as a radiological investigation to know the site of origin and tumor extension. In around 65% (n=18) cases, the tumor arose from the maxillary sinus; in 28% (n=8), it occurred from ethmoid sinuses; and in 7% (n=2) cases, it appeared from the frontal sinuses. In almost all cases, the tumors involve nearby sinuses and adjacent structures. In our series, four (14%) patients received only palliative treatment because of advanced disease at the time of presentation. Six (21%) patients had only surgery, and eighteen (64%) had both surgery and received postoperative radio or chemotherapy. Endoscopic resection was done in 15 (62.5%) patients, and combined open and endoscopic approaches were used in 9 (37.5%) patients. The most common histological variant was squamous cell carcinoma (n=8, 28%) (Table 3), followed by sinonasal undifferentiated carcinoma (SNUC) (n=6, 22%), Adenocarcinoma (n=3, 11%) and sarcoma (n=3, 11%). Recurrence was noticed in two patients; two patients died during follow-up, and one patient got maggot infection.

**Table 2:** Signs and symptoms of patients with malignancy of nose and paranasal sinuses

Sign and Symptoms	No. of patients (n) and their Percentage (%)
Facial or cheek swelling	15 (54%)
Nasal obstruction	12 (43%)
Diplopia	12 (43%)
Watering from eye	8 (28%)
Headache	7 (25%)
Epistaxis	6 (21%)
Nasal Discharge	5 (18%)
Swelling in hard palate	5 (18%)
Proptosis	4 (14%)
Loss of teeth or loose tooth in upper alveolus	4 (14%)
Loss of vision	2 (7%)
Ulceration over hard palate	2 (7%)
Ulceration over facial skin	2 (7%)

**Table 3:** Histological types of malignancy of nose and paranasal sinuses

Histological type	No. of patients (n) and their Percentage (%)
Squamous cell carcinoma	8 (28%)
Adenocarcinoma	3 (11%)
Sarcoma	3 (11%)
Olfactory neuroblastoma	1 (3%)
Plasmacytoma	2 (7%)
Lymphoma	1 (3%)
Malignant melanoma	1 (3%)
Myofibroblastic tumour	1 (3%)
Poorly differentiated/undifferentiated carcinoma	6 (22%)
Total	28 (100%)

## Discussion

Malignancy of paranasal sinuses is rare yet difficult to manage because of late presentation and the proximity to vital structures. Diagnosis is mainly delayed because of a lack of early specific symptoms, which are often neglected or ignored by patients thinking of benign diseases. Here, we analyze various presentations and management of different sinonasal malignancies. In the current study, the male-to-female ratio is 1.8: 1, and the mean age of presentation was 50.5 years. Similar findings of age distribution and sex ratio were also supported by other studies [6-8]. Jain S et al. [9] also suggest that the mean age of presentation for squamous cell carcinoma is 65.35 years compared to 59.63 years for adenocarcinoma. Facial swelling was the most common presentation in our series (54%. n=15), followed by nasal obstruction and diplopia (43%, n=12). It was in contrast to other series where nasal obstruction and nasal discharge were the most common presentation [10]. In one study, facial swelling was the most common presentation [11]. It might be due to the maximum number of tumors that arise from the maxillary sinus in our series (28%, n=8). In another study in Nigeria [12], epistaxis and nasal obstruction were the most common presentation. In our series, 75% (n=21) patients had the habit of chewing tobacco, and 57% (n=16) patients were smokers. Various studies [13,14] have the opinion that tobacco use leads to the development of squamous cell carcinoma of the nose and paranasal sinuses, while there is no association between adenocarcinoma and undifferentiated carcinoma. As per occupation, most of our patients were farmers (n=15, 54%), followed by homemakers (n=8, 28%). Various studies and systemic reviews [15,16] have strongly supported the association of certain professions like carpentry, forestry, woodworking, farming, and construction to sinonasal malignancies. Adenocarcinoma was the common variant associated with this. All our patients belong to lower socioeconomic groups. Though there was no direct evidence, many studies [17,18] have the opinion that social disparity, insurance status, and ethnicity influence survival in sinonasal carcinomas. In our study, the maxillary sinus was the most commonly involved sinus (n=18, 65%), followed by the ethmoid sinus (n=8, 28%) and frontal sinus (n=2, 7%). Studies by Poursadegh M et al. [11] and Gołabek W et al. [19] have a similar opinion. However, in some studies [14], the ethmoid sinus was the most common site, followed by the nasal cavity. According to Patel NN et al. [20], the nasal cavity was the preferred site for sinonasal malignancies. Depending on the clinical situation, magnetic resonance imaging (MRI) and computed tomography (CT) may be utilized separately or in combination. CT is useful for assessing erosion and bone landmarks and is crucial for using image guidance in surgery [21]. When it comes to invasion of the orbit, intracranial structures (dura, brain, cavernous sinus), perineural dissemination, infratemporal fossa, and face soft tissues, magnetic resonance imaging (MRI) is useful for differentiating soft tissues. All our patients had a CT scan or MRI for radiological investigations.

Endoscopic surgery was the preferred approach in our series (n=15, 62.5%). As per Ferrari M et al. [22], endoscopy is the preferred surgical approach except when there is the involvement of hard palate, skin, nasal bones, orbital roof, supraorbital dura, orbital muscles, etc., where open resection or a combination can be done. Squamous cell carcinoma was the most common histologic variant (28%, n=8), followed by sinonasal undifferentiated carcinoma (22%, n=6) in our series. Lots of studies [10,14,17] also support the claim that squamous cell carcinoma was the most common variant, followed by adenocarcinoma. In our series, sinonasal undifferentiated carcinoma was the second variant, possibly because of a small sample size and dealing with referral cases from other centers. Although rare, sarcoma was seen in 11% (n=3) of our patients. Sarcomas comprise 7% of all head and neck sarcomas, and involvement of the paranasal sinus has the worst prognosis among all sarcomas [23].

Limitation

Our study is a retrospective study with limited sample size. There is a definite lack of data because of incorrect documentation. There is a lack of scope to study the impact of other variables that may impact the disease progression and, subsequently, its management.

## Conclusions

Malignancy of the nose and paranasal sinuses is very rare. They presented with varied masked clinical presentations of benign diseases. Certain occupations have the highest risk of getting this disease. In our series, farmers comprise the highest percentage to have this. All of our patients belong to a lower socioeconomic group. Early identification and high clinical suspicion, along with imaging studies, are pivotal in managing malignancy of the nose and paranasal sinuses.

## References

[1] Shirazi N, Bist SS, Selvi TN, Harsh M (2015). Spectrum of sinonasal tumors: a 10-year experience at a tertiary care hospital in North India. Oman Med J.

[2] Rokade V, Shinde KJ, More GR (2020). Clinicopathological profile of sinonasal masses-a tertiary care centre study in rural India. Int J Otorhinolaryngol Head Neck Surg.

[3] Carsuzaa F, Favier V, Ferrari M (2023). Need for close interdisciplinary communication after endoscopic endonasal surgery to further personalize postoperative radiotherapy in sinonasal malignancies. Front Oncol.

[4] Goncalves N, Burnell LA, Motakef S, Modi PC (2016). Primary ethmoid sinus squamous cell carcinoma in a young adult man. S Afr Med J.

[5] Danesh-Sani SA, Sarafraz A, Chamani M, Derakhshandeh H (2016). Paranasal sinuses malignancies: a 12-year review of clinical characteristics. Med Oral Patol Oral Cir Bucal.

[6] Turner JH, Reh DD (2012). Incidence and survival in patients with sinonasal cancer: a historical analysis of population-based data. Head Neck.

[7] Koivunen P, Makitie AA, Back L, Pukkila M, Laranne J, Kinnunen I (2012). A national series of 244 sinonasal cancers in Finland in 1990-2004. Eur Arch Otorhinolaryngol.

[8] Poetker DM, Toohill RJ, Loehrl TA, Smith TL (2005). Endoscopic management of sinonasal tumors: a preliminary report. Am J Rhinol.

[9] Jain S, Li Y, Kuan EC, Tajudeen BA, Batra PS (2019). Prognostic factors in paranasal sinus squamous cell carcinoma and adenocarcinoma: a SEER database analysis. J Neurol Surg B Skull Base.

[10] Alabi BS, Afolabi OA, Omokanye HK, Dunmade AD, Ayodele SO (2017). Clinical presentation and outcome of sinonasal tumors in a Nigerian tertiary hospital - 6-year review. Niger Med J.

[11] Poursadegh M, Poursadegh F, Esmaeili M, Bakhshaee M (2015). Epidemiological survey of sinonasal malignancy in North-East Iran. Iran J Otorhinolaryngol.

[12] Fasunla AJ, Lasisi AO (2007). Sinonasal malignancies: a 10-year review in a tertiary health institution. J Natl Med Assoc.

[13] Hayes RB, Kardaun JW, de Bruyn A (1987). Tobacco use and sinonasal cancer: a case-control study. Br J Cancer.

[14] Bracigliano A, Tatangelo F, Perri F (2021). Malignant sinonasal tumors: Update on histological and clinical management. Curr Oncol.

[15] Binazzi A, Ferrante P, Marinaccio A (2015). Occupational exposure and sinonasal cancer: a systematic review and meta-analysis. BMC Cancer.

[16] Mofidi A, Tompa E, Kalcevich C (2022). Occupational exposure to wood dust and the burden of nasopharynx and sinonasal cancer in Canada. Int J Environ Res Public Health.

[17] Jafari A, Shen SA, Qualliotine JR (2021). Socioeconomic factors affect presentation stage and survival in sinonasal squamous cell carcinoma. Laryngoscope.

[18] Sharma RK, Del Signore A, Govindaraj S, Iloreta A, Overdevest JB, Gudis DA (2022). Impact of socioeconomic status on paranasal sinus cancer disease-specific and conditional survival. Otolaryngol Head Neck Surg.

[19] Gołabek W, Drop A, Gołabek E, Morshed K (2005). Site of origin of paranasal sinus malignancies [Polish]. Pol Merkur Lekarski.

[20] Patel NN, Maina IW, Kuan EC (2019). Adenocarcinoma of the sinonasal tract: a review of the National Cancer Database. J Neurol Surg B Skull Base.

[21] Kumar J, Daga R, Pradhan G, Meher R (2023). Sinonasal inflammation or neoplasm: raise the red flagsǃ-a pictorial review. Indian J Radiol Imaging.

[22] Ferrari M, Orlandi E, Bossi P (2021). Sinonasal cancers treatments: state of the art. Curr Opin Oncol.

[23] Martin E, Radomski S, Harley E (2019). Sarcomas of the paranasal sinuses: an analysis of the SEER database. Laryngoscope Investig Otolaryngol.

